# Immunomodulation with M2 macrophage–derived extracellular vesicles for enhanced titanium implant osseointegration under diabetic conditions

**DOI:** 10.1016/j.mtbio.2024.101385

**Published:** 2024-12-05

**Authors:** Yuzhao Cheng, Xin Dong, Jing Shi, Guangsheng Wu, Pei Tao, Nan Ren, Yimin Zhao, Fenglan Li, Zhongshan Wang

**Affiliations:** a The Stomatology Department of Shanxi Provincial People Hospital, Shanxi Medical University, Taiyuan, Shanxi, 030001, China; b State Key Laboratory of Oral & Maxillofacial Reconstruction and Regeneration, National Clinical Research Center for Oral Diseases, Shaanxi Key Laboratory of Stomatology, Department of Prosthodontics, School of Stomatology, The Fourth Military Medical University, China; c Department of Orthopedic Surgery, Tangdu Hospital, Air Force Military Medical University, Xi'an, Shaanxi Province, China; d Department of Stomatology, Qingdao Special Servicemen Recuperation Center of PLA Navy, No.18 Yueyang Road, Qingdao, 266071, China; e College of Chemistry and Bio-engineering, Yichun University, Yichun, Jiangxi, 336000, China

**Keywords:** Ti implants, Diabetes mellitus, Osseointegration, Macrophage, NLRP3 inflammasome

## Abstract

M2 macrophage–derived extracellular vesicles (M2-EVs) demonstrate the capacity to reduce pro-inflammatory M1 macrophage formation, thereby restoring the M1–M2 macrophage balance and promoting immunoregulation. However, the efficacy of M2-EVs in regulating macrophage polarization and subsequently enhancing osseointegration around titanium (Ti) implants in patients with diabetes mellitus (DM) remains to be elucidated. In this study, Ti implants were coated with polydopamine to facilitate M2-EVs adherence. In vitro experiment results demonstrated that M2-EVs could carry miR-23a-3p, inhibiting NOD-like receptor protein3(NLRP3) inflammasome activation in M1 macrophage and reducing the levels of inflammatory cytokines such as IL-1β by targeting NEK7. This improved the M1–M2 macrophage balance and enhanced mineralization on the Ti implant surfaces. The in vivo experiment results demonstrated that in diabetic conditions, the nanocoated M2-EVs significantly promoted high-quality bone deposition around the Ti implants. The current results provide a novel perspective for simple and effective decoration of M2-EVs on Ti implants; clinically, the method may afford osteoimmunomodulatory effects enhancing implant osseointegration in patients with DM.

## Graphical abstract

**Figure:**
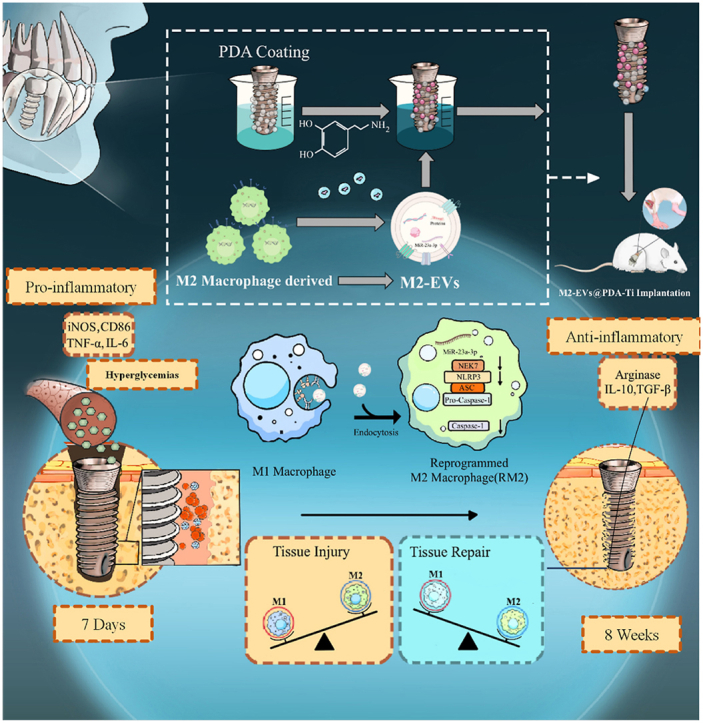

## 1 Introduction

Diabetes mellitus (DM), which worsens the inflammatory state of the patient's body, is often considered a contraindication for titanium (Ti) implantation [1]. Inflammatory responses are crucial for bone formation; however, persistent inflammation can impede bone formation around implants, which leads to instability, followed by potential implant failure [2].

Macrophages, which constitute the primary immune cells present in the vicinity of Ti implants [3,4], can be categorized into diverse subpopulations. These include classically activated macrophages (M1), alternatively activated macrophages (M2).However, the classification of macrophages is no longer confined to the M1 and M2 types. Other macrophage populations, such as tumor-associated macrophages (TAMs), CD169+ macrophages, and TCR + macrophages, have been recognized for their presence and significance under various pathological conditions [5,6].During the initial stage of implant placement under normal conditions, M1 macrophages play an indispensable role in the early inflammatory response [7]. These macrophages secrete pro-inflammatory cytokines and chemokines, which not only facilitate the clearance of pathogens by M1 macrophages but also promote the polarization of macrophages towards the M2 phenotype [8].The dynamic state between M1 and M2 macrophages is maintained. However, in hyperglycemic conditions, the function of M1 macrophages becomes compromised, resulting in a prolonged pro-inflammatory phase. This imbalance in the M1-M2 macrophage ratio can have detrimental effects on peri-implant bone formation. In this situation, one strategy involves converting chronic wounds into acute wounds, aiming to rejuvenate M1 macrophages in diabetic wounds and enable spontaneous polarization towards M2. An alternative strategy for addressing long-term non-healing wounds is to identify a switch that directly targets the M1/M2 transition, such as the use of M2-EVs [9,10].

Currently, exosome-based techniques are associated with some limitations, including short half-lives and instability, in vivo. Moreover, free exosomes administered directly may not be retained at the wound locations for a sufficient period and thus may not produce considerable benefits at the administration site [11]. As a catechol, dopamine is suitable as a binder for inorganic surface coatings. Similarly, catechol-rich poly(dopamine) exhibits robust molecular adhesion on virtually all types of substrates [12,13].

Recent studies have suggested that elevated glucose and islet amyloid polypeptide levels may indicate NOD-like receptor protein 3 (NLRP3) inflammasome activation in immune cells [14]. NLRP3 inflammasome activation potentially drives M1 polarization of macrophages, thereby compromising bone formation. On NLRP3 inflammasome activation, oligomeric complexes form with apoptosis-associated speckle-like protein containing a CARD(ASC) and caspase-1; these complexes attract M1 macrophages and release IL-1β and IL-18, promoting cell debris phagocytosis and bone resorption. Chen et al. demonstrated that reducing the number of M1 macrophages can significantly alter the bone immune microenvironment which leads to accelerated fracture healing in patients with DM [15]. Therefore, strategically managing macrophage homeostasis at implant interfaces is crucial to achieving the aforementioned objectives and enhancing implant osseointegration. Nevertheless, the specific function and process through which M2 macrophage–derived EVs (M2-EVs) improve the osseointegration of Ti implants in patients with DM have yet to be determined [16].

**Scheme 1:**
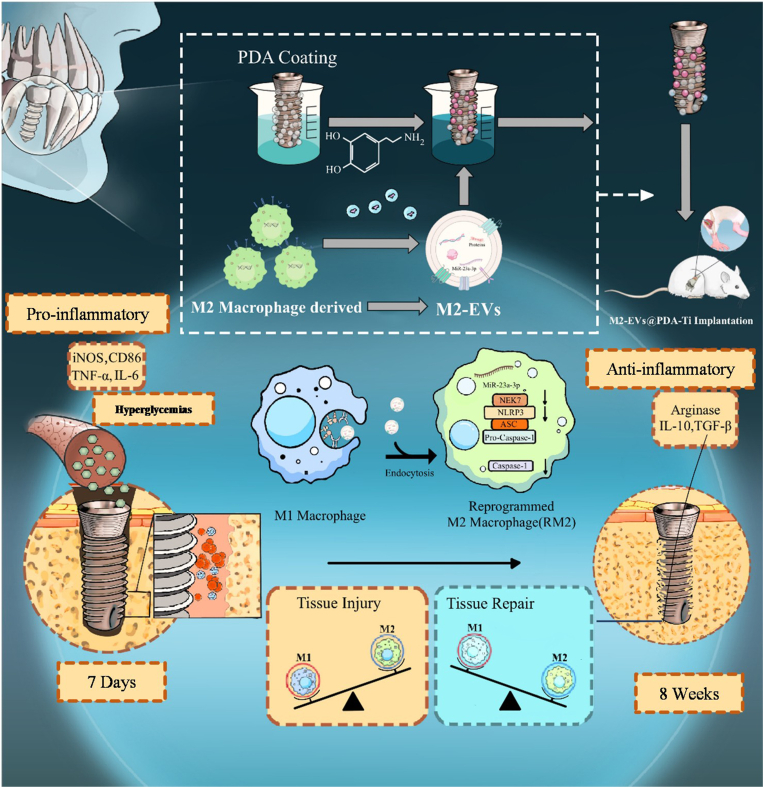
M2-EVs, as immunomodulators, reverse M1–M2macrophage imbalance and promote peri-implant osseointegration under diabetic conditions.

In this study,Ti-surface modification strategy involving M2-EVs deposition onto polydopamine (PDA)-coated Ti implant (PDA-Ti) surfaces to afford self-contained immunotherapy for enhancing implant osseointegration. First, the specific mechanism was investigated by which M2-EVs converted M1 macrophages into reprogrammed M2 macrophages. Subsequently, we examined the effects of M2-EVs-modified implants on bone formation in the surrounding areas. As illustrated in Scheme 1,the M2-EVs-modified implants could regulate osseointegration around implants in hyperglycemic conditions through a bone immunology perspective. Our results provide theoretical support and potential clinical treatment options for individuals with DM undergoing implantation procedures.

## 2 Materials and methods

### 2.1 Cell preparation and culture

Bone marrow–derived mesenchymal stem cells (BMSCs) were extracted by flushing the bone marrow from the femurs and tibias of 2-week-old male Wistar rats with phosphate-buffered saline (PBS; HyClone, USA). Next, the BMSCs were cultivated in α-minimum essential medium (Gibco, USA) supplemented with 10 % (v/v) fetal bovine albumin (HyClone, USA) and 1 % (v/v) penicillin–streptomycin (HyClone, USA; Fig. S1).

Bone marrow–derived macrophages (BMDMs) were obtained by entirely flushing out bone marrow from the femurs and tibias of 2-week-old male Wistar rats and culturing in high-glucose Dulbecco's modified Eagle medium (Gibco, USA) supplemented with 50 ng/mL of macrophage colony-stimulating factor (Peprotech, USA) for 7 days.Bone marrow-derived macrophages (BMDMs) were cultivated and subsequently converted into M1 macrophages through treatment with a high-glucose DMEM medium supplemented with 100 ng/mL lipopolysaccharide (LPS, bacterial source: *Escherichia coli*, Sigma, Germany, L2880) and 2.5 ng/mL interferon-gamma (IFN-γ, Peprotech, USA). This treatment was performed to mimic the diabetic mellitus (DM) microenvironment.All cells were maintained at 37 °C in a humidified environment under 5 % CO_2_, as reported previously [22].

### 2.2 PDA-Ti fabrication

In this study, grit-blasted and acid-etched grade 4 titanium implants manufactured by Kontour Medica (Xi'an, China) were utilized. These implants have a length of 5 mm and a diameter of 2 mm.Before PDA coating, the Ti implants were thoroughly cleaned through ultrasonication in ethanol and distilled water and then placed in a dopamine solution (2 mg/mL dopamine in 10 mM tris buffer, pH 8.5). Dopamine monomers were allowed to undergo self-initiated polymerization, resulting in the formation of a PDA layer on the surface of each implant. Subsequently, the polymerization reaction was carried out in the dark at room temperature for 24 h, following an optimized method provided by Qiu et al. [17].The binding between PDA and EVs was primarily realized through non-covalent bonds, including hydrogen bonding,π-π stacking, and electrostatic interactions [18].In this preparation, negatively charged polydopamine bound to EV surface molecules through electrostatic interactions (Fig. S4).After the completion of the reaction, all Ti implants were rinsed with distilled water to remove all unbound dopamine monomers. Finally, all resulting PDA-Ti implants were dried at 37 °C and stored until further use.

### 2.3 M2-EVs loaded PDA-Ti preparation

M2 macrophages were generated by treating RAW264.7 cells with 50 ng/mL IL-4 (Peprotech, China) in a medium supplemented with exosome-free serum for 48 h. The characterization of M2 macrophages was performed using flow cytometry (Fig. S2). In each experimental group, 10∗10-cm dishes, each containing 10^6 differentiated M2 macrophages cells, were cultured in 10 mL medium for 24 h. At this point, the supernatant was collected for further processing. The collected supernatant underwent sequential centrifugation steps: initially at 300g for 10 min, followed by 2,000g for 10 min, and finally at 10,000g for 30 min. To isolate only the M2-EVs, the supernatant was subjected to two additional centrifugation steps at 110,000g and 4 °C, each lasting 70 min (Optima XPN-100 Swinging-Bucket ROTOR, USA).All the extracted M2-EVs were stored at −80 °C until further use.

We examined the morphology, concentration, and size distribution of the M2-EVs under a transmission electron microscope (TEM; G2 Spirit Biotwin, Tecnai, USA) and NanoSight (Malvern, UK). M2-EVs surface expression of certain tagged proteins was identified through Western blotting.

The M2-EVs (100 μg/mL) and PDA-Ti were incubated together at 4 °C overnight to obtain M2-EVs-loaded PDA-Ti (M2-EVs@PDA-Ti); any excess solution was removed after incubation. Here, we used a previously reported methodology [19].

### 2.4 M2-EVs@PDA-Ti characterization

The surface morphology of M2-EVs@PDA-Ti and uncoated PDA-Ti was observed under a scanning electron microscope (SEM; HITACHI-4800; Hitachi, Japan). Surface contact of the samples were tested using a contact angle tester (Kruss, Germany). The three-dimensional morphology and roughness of all samples were detected on an atomic force microscope (AFM) in the tapping mode.

### 2.5 Cellular uptake of M2-EVs

DiI-labeled M2-EVs were seeded onto macrophages in a confocal dish (Wuxi NEST Biotechnology, China), followed by incubation for 4h,24h to allow for M2-EVs uptake. Next, the cells were washed with PBS three times and then fixed in 4 % paraformaldehyde (PFA; Solarbio, China) for 15 min.The location of M2-EVs was captured under the laser-scanning confocal microscope (A1+/A1R+; Nikon, Tokyo, Japan).

### 2.6 Western blotting

Cells were lysed using RIPA Lysis Buffer (Gibco, Montclair, USA) on ice. Next, the mixture was centrifuged at 12,000g,4 °C for 15min. Proteins in the supernatant were quantified using a bicinchoninic acid protein assay (Beyotime, China).The membrane was incubated with primary antibodies against the following proteins at 4 °C overnight.The membrane was carefully rinsed three times with tris-buffered saline with 0.1 % Tween 20 at room temperature and then incubated with the secondary antibody SA00001-2 (Proteintech, China) for 1h. Protein bands were visualized using a chemical discharge imaging system (Bio-Rad, United States). The gray values of the target proteins and GAPDH were determined using ImageJ (New York, USA).The target protein expression was normalized using that of GAPDH.The antibody dilutions were listed in Supplementary Table S2.

### 2.7 Quantitative reverse transcription polymerase chain reaction

Total RNA was extracted using RNAiso Plus (Takara, Japan), and it was reverse-transcribed to cDNA by using PrimeScript RT Master Mix (Takara, Japan). The cDNA was then subjected to quantitative reverse transcription polymerase chain reaction (qRT-PCR) by using TB Green Premix Ex TaqTMII (Takara, Japan). For mRNAs and miR-23a-3p, we used *GAPDH* and *U6* as the internal controls, respectively. Supplementary Table S1 lists all primer sequences for qRT-PCR; all the primers were prepared by Accurate Biotechnology (Hunan, China).

### 2.8 EdU assay

We performed the EdU assay from Beyotime (China), according to the manufacturer's instructions. In brief, the cells were incubated with a 1 × EdU working solution for 4 h and then fixed with 4 % formaldehyde. Next, the fixed cells were labeled using a click reaction cocktail, and their nuclei were counterstained with Hoechst. Finally, the samples were visualized under an Eclipse fluorescence microscopy (Nikon).

### 2.9 Transwell assay

We assessed cell migration in a transwell system (Corning, NY, USA) equipped with 6.5-mm and 8.0-μm pore polycarbonate membrane inserts. In the bottom chamber of this system, we added 600 μL of RM2/M2 conditioned medium, whereas we added 200 μL of BMSCs suspended in serum-free medium in its top chamber. Next, the system was placed in a humidified incubator for 24 h. The BMSCs were then fixed with 4 % PFA. The cells in the top chamber were removed using a cotton swab, and the migrated cells in the bottom chamber were stained with crystal violet for 30 min. Stained cells in five randomly selected fields were enumerated under an inverted microscope (Olympus, Tokyo, Japan).

### 2.10 Cell counting kit 8 assay

We seeded BMSCs in 96-well plates at 3000 cells per well and incubated them at 37 °C under 5 % CO_2_. After incubation, the cells were treated with 10 μL of cell counting kit 8 (CCK8) reagent (Gipbio, Montclair, USA) at 37 °C for 0–48 h. The optical density of each cell was measured at 450 nm on a microplate reader (Spectramax, USA), and the percentage of viable cells was calculated.

### 2.11 Alkaline phosphatase and alizarin red S staining

We co-cultured RM2 macrophages and BMSCs in α-minimum essential medium for a predetermined duration and then stained cells in different growth phases for alkaline phosphatase (ALP) activity and with alizarin red S (ARS).

For ALP staining, the cells were fixed with 4 % PFA for 15 min on postculture day 7. We then stained the cells by using the BCIP/NBT ALP Color Development Kit (Beyotime) and quantified the developed color using an ALP assay kit.

For ARS staining, the cells were fixed with 4 % PFA for 15 min on postculture day 14. We then subjected the cells to ARS (Beyotime) for approximately 20 min, followed by three washings with dH_2_O, and then by incubation at room temperature for another 20 min until the color developed. Finally, deionized water was added to halt further color development. All stained cells were observed under an inverted microscope (Olympus, Tokyo, Japan). For quantification, we used a multifunctional enzyme marker (BioTek, USA) and determined the absorbance of the stained cells at 562 nm.

### 2.12 Immunofluorescence analysis

We first incubated cells with primary antibodies against iNOS and Arg-1 (arginase-1) at 4 °C for 14 h. Next, the cells were incubated with one of the following secondary antibodies at room temperature for 2 h: goat antirabbit immunoglobulin G (H + L) or FITC-conjugated goat antimouse immunoglobulin G (H + L). Daphne blue staining was used to stain nuclei. Finally, fluorescence cells were identified under an A1+/A1R + laser-scanning confocal microscope (Nikon, Tokyo, Japan).

### 2.13 Animal surgical procedures

All experiments involving animals were approved by the Ethics Committee of the School of Stomatology, The Fourth Military Medical University (approval no.kq-051). Female Wistar rats (age = 2 months) and GK rats (age = 3 months) were randomly assigned to one of two groups. The experiment comprised three groups (n = 6 per group): (1) Wistar rats implanted with PDA-Ti, (2) GK rats implanted with PDA-Ti, and (3) GK rats implanted with M2-EVs@PDA-Ti.

The implantation procedure was performed according to a protocol described previously [23]. In brief, We anesthetized all rats using 1 % sodium pentobarbital solution at 45 mg/kg body weight. Next, by using a standard sterile animal surgical technique, we placed the implants in the femoral mid-shaft of the rats. To prevent hypothermia, we employed heating pads. At 8 weeks after implantation, the rats were euthanized, and their femur radiographs and histological samples were obtained.

### 2.14 Microcomputed tomography analysis

Histological specimens from the implanted femurs were fixed in 4 % PFA overnight. Osteointegration around the implant was examined on a micro-computed tomography (micro-CT) instrument (Siemens Inveon, Erlangen, Germany). Three-dimensional structures were generated from the regions of interest (ROI), located approximately 100 μm from the implant surface. The following morphometric characteristics of the bone surrounding the implants were examined: bone volume percentage (BV/TV), trabecular thickness (Tb.Th), trabecular number (Tb.N), and trabecular separation (Tb.Sp).

### 2.15 Van Gieson staining

Histological specimens from the implanted femurs were dehydrated in an alcohol gradient, embedded in methyl methacrylate. Next, we sliced the specimens into approximately sections parallel to each implant's long axis. These sections were polished and stained using a Van Gieson staining solution (1.2 % trinitrophenol and 1 % acid fuchsin). Finally, we qualitatively examined bone development in each section on a standard light microscope and analyzed the images by using Image-Pro Plus (Image-Pro, USA); we also calculated the bone-to-implant contact (BIC) ratio and bone area (BA).

### 2.16 Statistical analysis

We used GraphPad Prism 9.0 (GraphPad Software, San Diego, CA, USA) for all statistical analyses. All quantitative data were computed as the means and their standard deviations from at least three separate replicates. One-way analysis of variance was used to compare data between more than two groups, whereas the *t*-test was used to assess differences between two groups. *P* < 0.05 was considered to indicate statistical significance.

## 3 Results and discussion

### 3.1 M2-EVs@PDA-Ti preparation and characterization

**Fig. 1:**
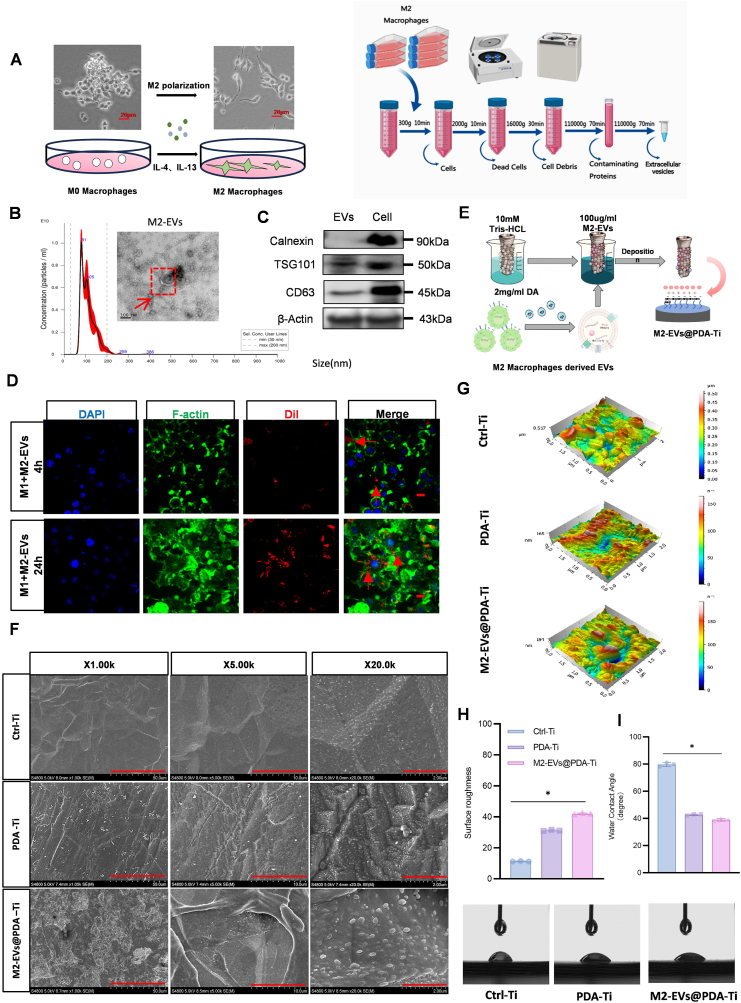
M2-EVs@PDA-Ti preparation and surface modification. (A) Schematic of M2-EVs isolation. (B) M2-EVs morphology visualized under a TEM (scale bar: 100 nm). M2-EVs size distribution examined through NTA. (C)Western blots for TSG101, CD63, and calnexin expression.(D)DiI-labeled M2-EVs uptake in M1 macrophages under a confocal microscope (scale bar: 20 μm). (E) Schematic of M2-EVs@PDA-Ti preparation.(F)Surface topography images of Ti, PDA-Ti, and M2-EVs@PDA-Ti (scale bar: 50, 10, or 2 μm). (G)Ti, PDA-Ti, and M2-EVs@PDA-Ti surfaces under an AFM.(H) Surface roughness values in AFM analysis.(I) Water contact angle images and statistical analysis. (n = 3,∗*P* < 0.05,∗∗*P* < 0.01).

By using IL-4, we activate RAW264.7 cells to obtain alternatively activated M2 macrophages (Fig. 1A). Then, M2-EVs were extracted from M2 macrophages by using differential centrifugation. Under a TEM, the extracted M2-EVs appeared as circular vesicles with double-layered membranes. Through nanoparticle tracking analysis (NTA) with a size range of 30–200 nm and at 4.91E+11 particles/mL,we determined the average M2-EVs particle size to be 111.1 ± 0.7 nm (Fig. 1B). Western blot analysis revealed that the M2-EVs bore TSG101 and CD63 without calnexin an endoplasmic reticulum marker (Fig. 1C). The results showed that M1 macrophages could capture M2-EVs, as indicated by DiI-labeled M2-EVs noted in the cytoplasm near the nuclei (Fig. 1D). As such, the M2-EVs demonstrated effective separation, and M1 macrophages could internalize them.

To enhance the adherence of M2-EVs on Ti implant surfaces, a PDA coating was applied to the implants. PDA possessed the ability to adhere to various types of organic and inorganic materials and form a coating on surfaces [20].The stability of the modifier was crucial for positioning an implant surface, and the PDA coating was necessary to help anchor the EVs. Since PDA itself is negatively charged (−30.2 mV) and the membrane surface of M2-EVs had a potential of −4.3 mV, the two could combine through electrostatic adsorption (Fig. 1E). Zeta potential measurements showed significant changes in the M2-EVs@PDA (−13.3 mV) composite material (Fig. 4SA). After loading PDA-Ti with M2-EVs, we assessed the modified surfaces in each group. Under the SEM, the surface topography of M2-EVs@PDA-Ti and uncoated PDA-Ti. The results demonstrated that the M2-EVs were uniformly formed and distributed on PDA-Ti surfaces (Fig. 1F). AFM images taken with a height sensor revealed that Ti implant surface roughness was high in the PDA group and the M2-EVs@PDA-Ti (Fig. 1G and H). In the water contact angle images, the PDA-Ti and M2-EVs@PDA-Ti surfaces demonstrated higher hydrophilicity than the control implants (Fig. 1I).

Most existing biomaterial surface modification strategies have involved chemical synthesis and inflexible fixation. However, some of these methods have limited the effectiveness of EVs modification, including (i) achieving local and sustained drug release, (ii) enhancing bioactivity, and (iii) ensuring safety [21,22].By detecting the encapsulation rate and release rate of both, the results demonstrated that compared to Ti without PDA coating, a large amount of M2-EVs could bind to the Ti surface with PDA coating (Fig. S4B). Furthermore, to confirm that M2-EVs, as vesicles, could maintain stable biological activity, their size and polydispersity index (PDI) were monitored after long-term storage. The relatively constant size and polydispersity index (PDI) demonstrated satisfactory stability of the M2-EVs@PDA complexes over the investigated time period (Fig. S5). The biosafety of M2-EVs@PDA was thoroughly assessed through cell viability and dead staining assays, as well as hemolysis experiments. As depicted in Figs. S6 and S7, no significant apoptosis of macrophages was detected in either the M2-EVs or M2-EVs@PDA treatment groups, indicating excellent cell viability in both conditions, as evidenced by the predominant green fluorescence. Furthermore, the hemolysis rates of the M2-EVs and M2-EVs@PDA groups were determined to be only 1.68 % and 2.23 %, respectively, which strongly suggests that M2-EVs are highly biocompatible and preferred biomodulatory substances for implant surface modification. The innovative functionalized M2-EVs@PDA-Ti creation strategy developed in this study provides a flexible, reliable, and efficient approach for the modification of Ti implant surfaces. Collectively, this evidence suggests the successful construction of M2-EVs@PDA-Ti.

### 3.2 M1/M2 macrophage imbalance occurs during early stage of implant osseointegration under diabetic conditions

**Fig. 2:**
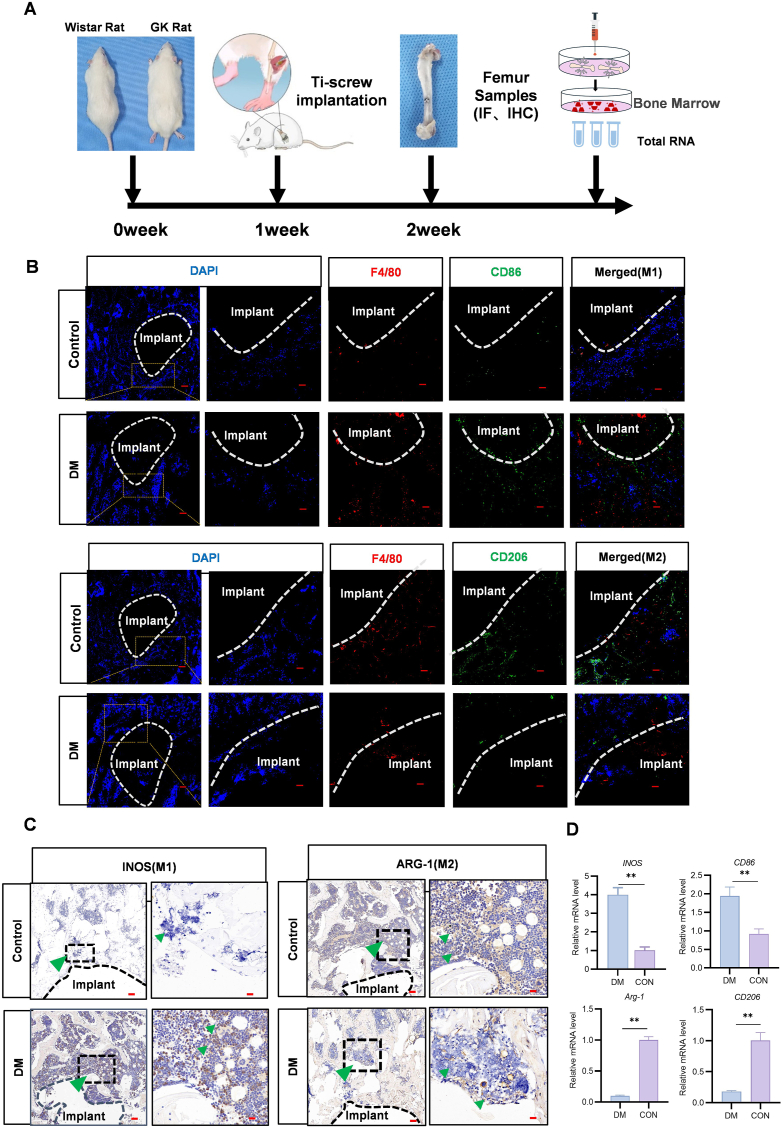
M1–M2 macrophages imbalance occurs during early stage of implant osseointegration under diabetic conditions. (A) Design and schedule of the in vivo study. (B) Immunofluorescence staining demonstrating M1 (F4/80+, CD86) and M2 (F4/80+, CD206) macrophages localization. In the DM group, M1 macrophages positivity was high, whereas that of M2 macrophages was low (scale bars: 200,50 μm). (C) Immunohistochemical staining showing elevated, sustained M1 and M2 macrophages (iNOS and ARG-1) positivity in the control and DM groups (scale bar: 100, 20 μm). (D) iNOS, CD86, ARG1, and CD206 mRNA levels (n = 3, *∗∗P < 0.01*).

DM is detrimental to peri-implant osteogenesis (Fig. S3). After Ti-implants were implanted, the biologically inert nature of the extrametallic material exacerbates a local immune response, which involves macrophage recruitment to the implant surface within hours of the implantation procedure, triggering a series of early inflammatory responses [3,4]. Under diabetic conditions, this local immune response demonstrated the predominance of M1 macrophages, which are proinflammatory. As such, we subsequently explored the in vivo effects of implantation of Ti implants in rats with DM (Fig. 2A). We used immunofluorescence and immunohistochemistry techniques to evaluate macrophage phenotypes around Ti implants over 7 post implantation days. The results showed that GK rats with the implant demonstrated more F4/80+/CD86 M1 macrophages than the healthy control rats. Moreover, immunohistochemistry indicated iNOS expression (indicative of M1 macrophages) was higher in GK rats with the implant than in healthy control rats (Fig. 2B and C). Furthermore, in qRT-PCR analysis, the expression of mRNA of the M1 macrophage marker genes iNOS and CD86 was significantly higher, whereas that of the M2 macrophage marker genes ARG1 and CD206 was significantly lower, in the surrounding tissues of the implant in the DM rats. The results showed that the iNOS (M1) and CD86 (M1) mRNA levels were significantly increased in DM rats with Ti implants, whereas the mRNA levels of CD206 (M2) and ARG1 (M2) were significantly lower (Fig. 2D). These results indicated that in the first stage after implantation, macrophages with proinflammatory phenotypes were dominant in DM [23,24]. Research has found hyperglycemia may alter the release and function of small extracellular vesicles (sEVs) in macrophages.These sEVs induce the secretion of extracellular matrix components and inflammatory factors, that can stimulate tissue inflammation and inhibit the process of autophagy [25].Because most chronic wounds result from prolonged inflammation lasting over 1 week, the M1 macrophages rapid conversion of early to M2 macrophages can effectively prevent wounds from becoming chronic [26,27].The exosomes isolated from alternatively activated M2 macrophages (M2-Exo) could induce direct reprogramming of M1 to M2 macrophages achieving extremely high conversion efficiency [28].Consequently, the mechanism by which the contents of M2-EVs promote the reprogramming of M1 macrophages was further investigated in this study.

### 3.3 M2-EVs inhibits NLRP3 expression in M1 macrophages via miR-23a-3p

**Fig. 3:**
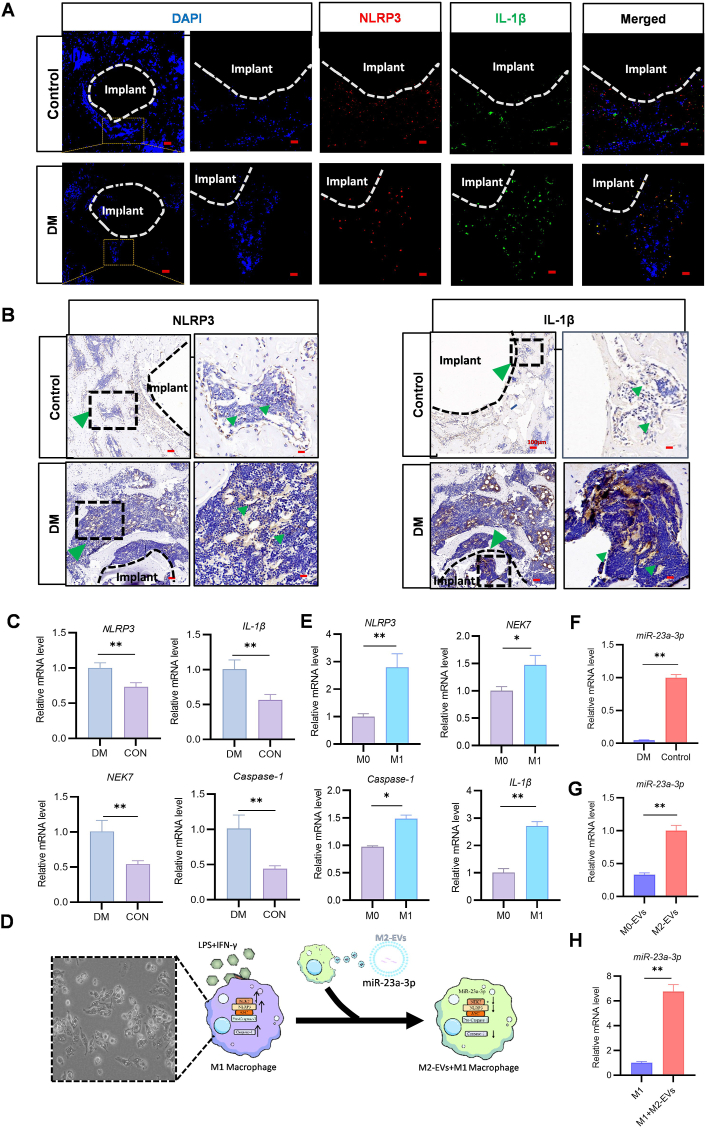
M2-EVs inhibit NLRP3 expression in M1 macrophages via miR-23a-3p. (A) Immunofluorescence staining revealing that IL-1β and NLRP3 expression was higher in the DM group than in the control group (scale bars: 200,50 μm). (B) Immunohistochemical staining demonstrating that IL-1β and NLRP3 expression was higher in the DM group than in the control group (scale bars: 100,20 μm). (C) NLRP3, NEK7, IL-1β, and Caspase-1 mRNA levels. (D) Schematic of the underlying mechanism. The morphology characteristic of the M1 phenotype is shown (scale bar: 50 μm). (E) qRT-PCR analysis revealing that NLRP3, IL-1β, NEK7, and Caspase-1 mRNA levels were higher in M1 macrophages than in M0 macrophages. (F) qRT-PCR analysis showing that miR-23a-3p expression in Ti implant–bone tissue samples was higher in healthy rats than in DM rats. (G) qRT-PCR analysis for miR-23a-3p expression in M0-EVs and M2-EVs. (H) qRT-PCR analysis for miR-23a-3p expression in M1 macrophages after M2-EVs uptake.(n = 3; ∗*P* < 0.05, ∗∗*P* < 0.01).

In previous experiments, the GK rats demonstrated a higher number of CD86, iNOS (M1) than the healthy control rats.In macrophages, NLRP3 are responsible for M1 polarization via the NLRP3/caspase-1/IL-1β pathway under high-glucose conditions [29]. NEK7,a mitogen activated protein kinase component, has a role in NLRP3 assembly and activation throughout the activation phase [30]. Similarly, we observed higher expression of NLRP3 and IL-1β in the tissues surrounding the Ti implants in the GK rats than in the healthy control rats (Fig. 3A and B). They also demonstrated significantly higher mRNA levels of NLRP3, NEK7, IL-1β, and caspase-1 (Fig. 3C). Hyperglycemia and prolonged inflammation in the implant microenvironment promoted macrophage polarization and inhibited BMSCs differentiation. Therefore, in subsequent experiments, we used a high-glucose medium supplemented with LPS and IFN-γ to mimic the DM microenvironment (Fig. 3D). We also used qRT-PCR to compare the levels of NEK7, NLRP3, caspase-1, and IL-1β in M1 macrophages (Fig. 3E); these results were identical to those obtained for the in vivo experiments.

To understand the mechanism through which M2-EVs reprogrammed M1 macrophages, we investigated micro-RNA expression in M2-EVs. The results demonstrated that miR-23a-3p negatively controls NEK7, preventing NLRP3 activation. MiR-23a-3p inhibits NEK7 expression by binding to the 3′untranslated region of NEK7 mRNA—consistent with other experimental findings [31]. In vivo experiments, miR-23a-3p levels were lower in the tissues surrounding the implants in the GK rats than in the healthy control rats (Fig. 3F). Nevertheless, M2-EVs demonstrated high levels of miR-23a-3p (Fig. 3G). Thus, we used M2-EVs to mitigate NLRP3 activation. Our qRT-PCR results demonstrated that co-administration of M2-EVs and M1 macrophages increased miR-23a-3p expression but reduced NEK7, NLRP3, caspase-1, and IL-1β expression (Fig. 3H).The corresponding protein expression levels remained identical (Fig. S8). These results suggested that M2-EVs alter the function of M1 macrophages by releasing miR-23a-3p, which lowers NLRP3 expression. Therefore, miR-23a-3p in M2-EVs potentially reprogram M1 macrophages.

### 3.4 M2-EVs promote reprogramming of M1 macrophages to RM2 macrophages in vitro

**Fig. 4:**
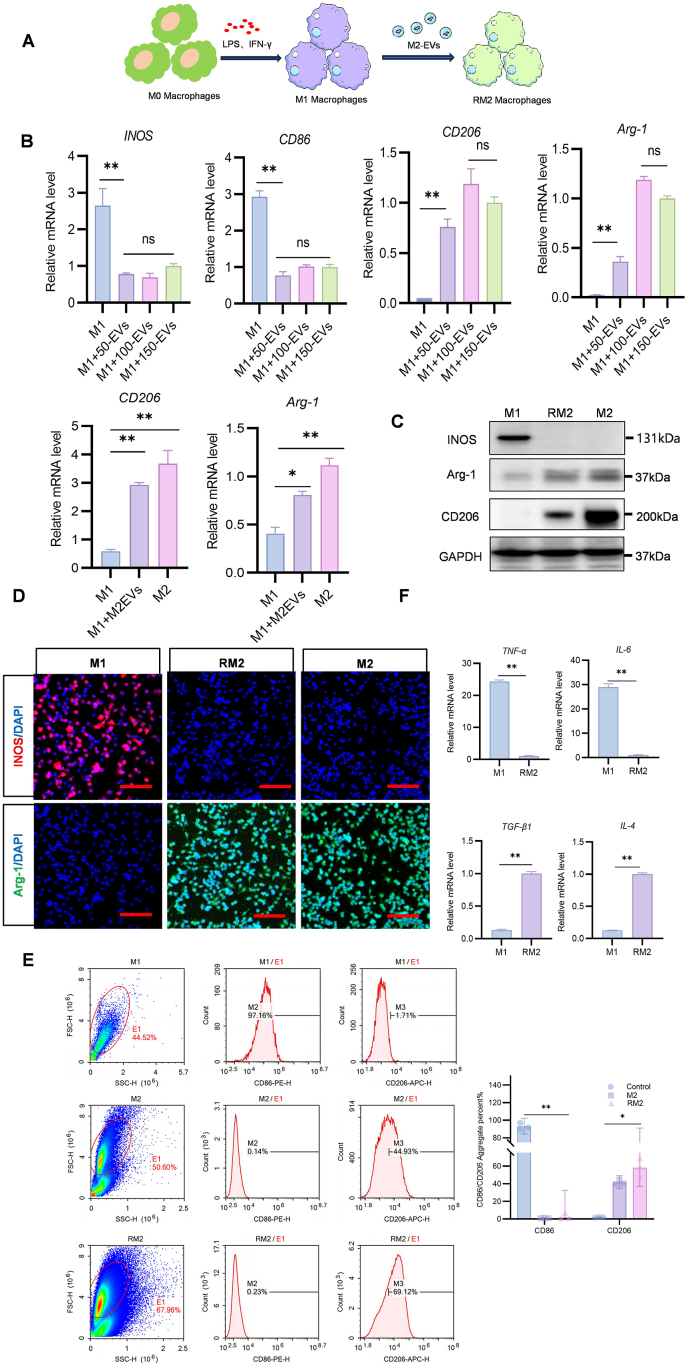
M2-EVs promote M1 macrophages reprogramming in vitro. (A) Schematic of M2-EVs promoting M1 macrophages reprogramming. (B) qRT-PCR analysis of CD206, Arg-1, iNOS, and CD86 mRNA levels in macrophages from groups treated with 50, 100, and 150 μg/mL M2-EVs. (C) Western blotting for expression of M1, M2, and RM2 macrophage markers, including iNOS, CD206, and Arg-1 levels. (D) Immunofluorescence analysis of M1, M2, and RM2 macrophage markers, including iNOS and Arg-1 (scale bar: 100 μm). (E) M1, M2, and RM2 macrophage co-localization based on CD86 and CD206 staining through flow cytometry. (F) qRT-PCR analysis of TNF-α,IL-6, TGF-β1 and IL-4 mRNA levels in M1 and RM2 macrophages (n = 3, ∗*P* < 0.05,*∗∗P < 0.01*).

Macrophage reprogramming is a crucial area of research in immunology [32].By modulating the inflammatory signaling pathways within macrophages and altering their polarization state, a transition from the M1 phenotype to the M2 phenotype can be induced [[33], [34], [35]].The surface components M2-EVs inherit from macrophage cell membranes provide them with high selectivity and molecular precision but low immunogenicity [36,37]. Thus, because of the membrane-bound proteins and lipids provided by the parent-cell, M2-EVs can specifically fuse with macrophages (Fig. 4A). To verify whether M2-EVs triggered the conversion of the M1 to M2 phenotype, we co-incubated different concentrations of M2-EVs (50, 100, and 150 μg/mL) with M1 macrophages in a serum-free medium for 24 h. Treatment with 50 μg/mL M2-EVs reduced iNOS and CD86 mRNA but increased CD206 and ARG1 mRNA levels, indicating that the M1 macrophages were reprogrammed into RM2 macrophages.To reduce reagent use, we used M2-EVs at a concentration of 100 μg/mL for further experimentation. We also noted that CD206 and ARG1 mRNA levels did not differ between modified RM2 and alternatively activated M2 macrophages (Fig. 4B). Because macrophages must maintain an anti-inflammatory state at all times to favor wound healing, the sustainability of the phenotypic switch guided by M2-EVs was investigated through Western blotting and immunofluorescence staining. The results demonstrated that RM2 macrophages expressed specific alternatively activated M2 macrophage markers, such as CD206 and ARG1 (Fig. 4C and D). Moreover, to precisely determine the degree of M1 reprogramming induced by M2-EVs in macrophages, we compared the CD86 and CD206 levels in M1, alternatively activated M2, and RM2 macrophages through flow cytometry; the results were identical to those noted through Western blotting. RM2 and alternatively activated M2 macrophages expressed similar amounts of CD206 (Fig. 4E). After M2-EVs uptake, M1 macrophages began releasing higher levels of repair-mediating cytokines such as IL-4 and TGF-β1 but lower levels of proinflammatory cytokines such as TNF-α and IL-6 compared with uninduced M1 macrophages (Fig. 4F). These results suggested that M2-EVs guide the phenotype switching in M1 macrophages.

### 3.5 RM2 macrophages promote osteogenesis on Ti surface

**Fig. 5:**
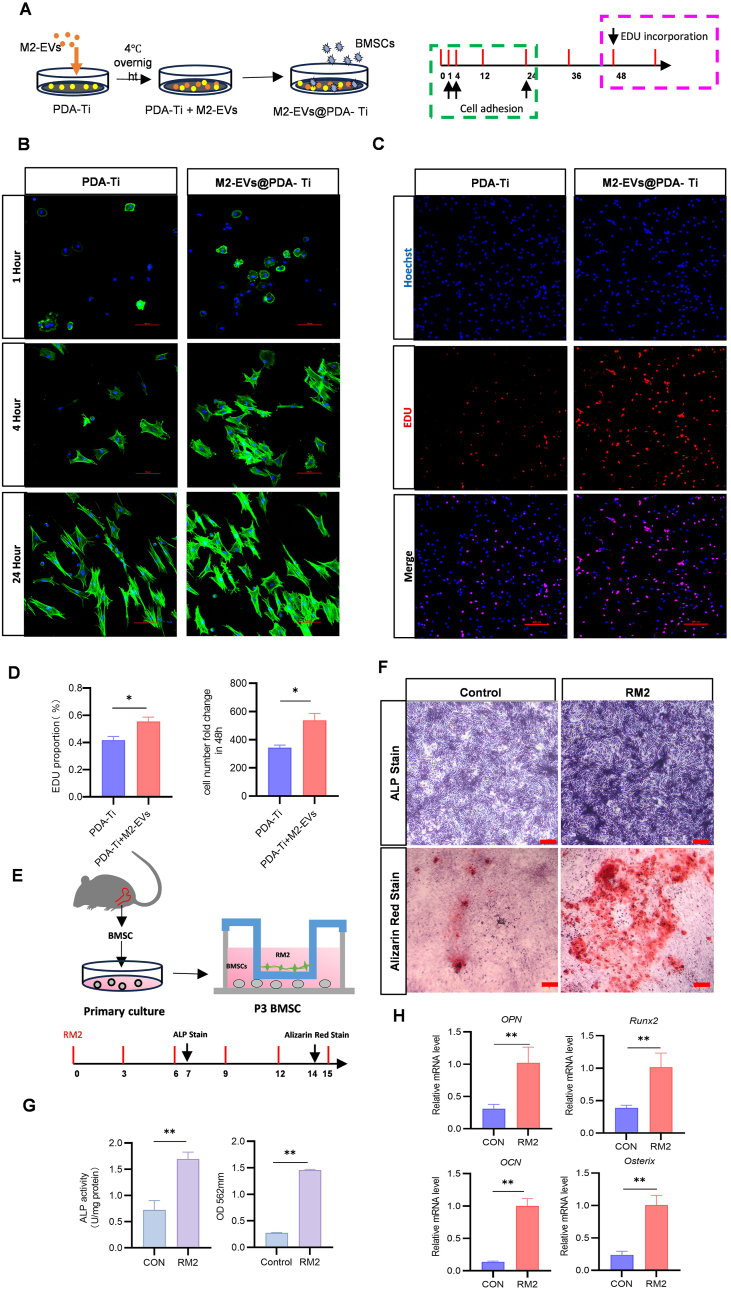
M2-EVs increase BMSCs adhesion on PDA-Ti surfaces and promote osteogenesis through RM2 macrophages. (A) Schematic of M2-EVs coating on PDA-Ti and co-culture with BMSCs. (B) Fluorescent images of BMSCs adhering on different samples after 1, 4, and 24 h (scale bar: 100 μm). (C) Confocal laser scanning microscopic images of osteoblasts after 48 h EdU staining. Cells are stained fluorescent red, whereas the nuclei are stained blue by Hoechst(scale bar: 200 μm). (D) EdU staining of BMSCs with corresponding quantitative analyses. (E) Schematic of the BMSCs–RM2 macrophages co-culture process. (F) Representative images of ALP and ARS staining of BMSCs after 7 and 14 days of continuous induction with RM2 macrophages supernatant, respectively (scale bars: 200 μm). (G) Quantitative analyses of ALP and ARS staining of BMSCs. (H) qRT-PCR analysis of OPN, RUNX2, OSX, and OCN mRNA levels in BMSCs treated with RM2 macrophages supernatant (n = 3,∗*P* < 0.05, *∗∗P < 0.0*1). (For interpretation of the references to color in this figure legend, the reader is referred to the Web version of this article.)

In the Ti implant–bone regeneration stage, BMSCs were noted to play crucial recruitment and adhesion roles in bones surrounding the Ti implants, significantly affecting subsequent bone regeneration (Fig. 5A). Fluorescence microscopy images demonstrated that when BMSCs were co-cultured with PDA-Ti or M2-EVs@PDA-Ti under identical high-glucose conditions, more BMSCs adhered to M2-EVs@PDA-Ti than to PDA-Ti at 1 h, with most of the adhering BMSCs being spherical or elliptical. At 4 and 24 h,BMSCs adhered to both PDA-Ti and M2-EVs@PDA-Ti; all these BMSCs were triangular or polygonal, with abundant branch-like and filamentous pseudopodia, tightly connected in a mesh-like pattern (Fig. 5B). In addition, the EdU assay results demonstrated that both PDA-Ti and M2-EVs@PDA-Ti led to significant cell proliferation at 48h, with the M2-EVs@PDA-Ti leading to a 10.27 % higher EdU positivity rate than in the control group (Fig. 5C and D). The immunomodulatory role of the macrophage phenotype was predominantly noted in the early and intermediate stages. The switch from the M1 to RM2 phenotype results in a suitable immune microenvironment facilitating peri-implant osteogenic differentiation and bone remodeling. We next co-cultured BMSCs with RM2 macrophages for 7 and 14 days (Fig. 5E) and evaluated them through ALP and ARS staining. The results demonstrated that ALP staining was stronger in the RM2 macrophage group than in the control group, indicating that BMSCs exhibit high ALP activity in the presence of RM2 macrophages. The ARS staining results, both qualitatively and quantitatively, aligned with the ALP staining findings: the RM2 macrophage group demonstrated more calcified patches, indicating higher osteogenic potential (Fig. 5F and G). Furthermore, the mRNA levels of signature genes related to osteogenesis and bone improvement (i.e., OPN, RUNX2, OSX, and OCN) were analyzed through qRT-PCR. Both RUNX2 and OPN typically reflect the early osteogenic differentiation capacity, whereas OCN and osterix generally reflect late osteogenic capacity [38]. The RM2 macrophage group exhibited significantly higher mRNA levels of these genes than the control group (Fig. 5H). These findings suggested that in DM mimicking micro-environments, M2-EVs@PDA-Ti promote osteogenesis in BMSCs through RM2 macrophages.

### 3.6 RM2 macrophages secrete TGF-β1 to enhance osteogenesis in BMSCs

**Fig. 6:**
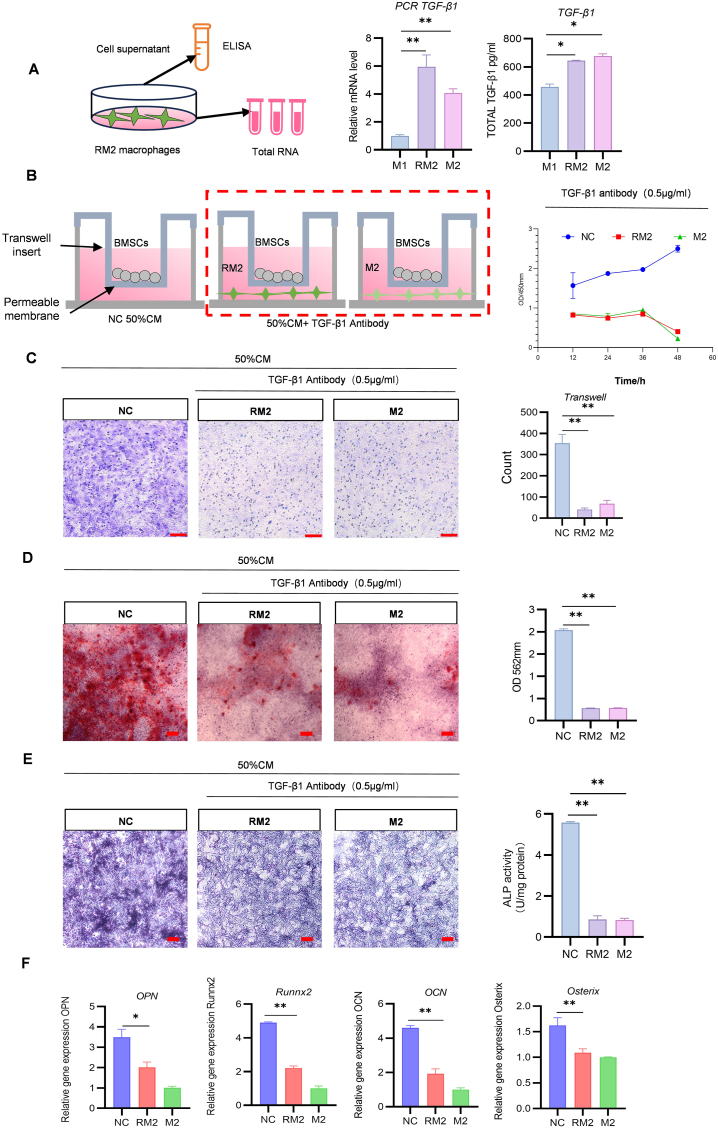
RM2 macrophages promote osteogenic differentiation of BMSCs via TGF-β1 secretion. (A) ELISA and qRT-PCR of TGF-β1 secreted by M1, RM2, and M2 macrophages. (B) Schematic of establishment of a cell-culture system using transwell chambers and treatment with antibodies against TGF-β1. BMSCs proliferation was assessed using the CCK-8 assay. (C) Effects of TGF-β1 antibodies on BMSCs vertical migration, indicated by three micrographs of BMSCs in separate groups at the bottom of transwell chamber (scale bar: 100 μm). (D)ARS staining for osteogenic differentiation of BMSCs after 14 days of co-culture (scale bar: 200 μm). (E)ALP staining for osteogenic differentiation of BMSCs after 7 days of co-culture (scale bar: 200 μm). (F) qRT-PCR analysis of OPN, RUNX2, OSX, and OCN mRNA levels in the BMSCs–RM2 macrophages co-culture system treated with TGF-β1 antibodies (n = 3; ∗*P* < 0.05, ∗∗*P* < 0.01).

In the bone formation stage, tissue remodeling is promoted by IL-4, IL-10, and TGF-β1 release from the alternatively activated M2 macrophages [39,40], in turn activating the osteogenic signaling pathways in BMSCs [41]. TGF-β1, a TGF-β family protein, has been investigated thoroughly because of its role in regulating cell proliferation and differentiation, wound healing, and the immune system. We measured TGF-β1 mRNA and protein levels in cell supernatants through qRT-PCR and ELISA, respectively. The results indicated that RM2 macrophages and the alternatively activated M2 macrophages secreted TGF-β1 at similar levels (Fig. 6A). To further confirm the involvement of TGF-β1 in the osteogenic promotion in RM2 macrophages, we co-cultured BMSCs with RM2 or alternatively activated M2 macrophages in transwell chambers in the presence of 0.5 μg/mL TGF-β1 inhibitor. After 12–48 h, the CCK8 assay results revealed that in both the RM2 and alternatively activated M2 macrophages groups, BMSCs proliferation was inhibited relative to the control group (Fig. 6B). The transwell assay results also demonstrated that the BMSCs migration ability was compromised by the TGF-β1 inhibitor (Fig. 6C). Similarly, the ALP and ARS staining results indicated that the TGF-β1 inhibitor hindered the osteogenic activity of BMSCs (Fig. 6D and E).In qRT-PCR of the osteogenic related genes OPN, RUNX2, OSX, and OCN, the results demonstrated that the mRNA levels of the genes significantly decreased in the RM2 macrophages and alternatively activated M2 macrophages than in the control group (Fig. 6F). These results suggested that RM2 macrophages promote BMSCs differentiation and osteogenesis through the TGF-β pathway.

### 3.7 M2-EVs@PDA-Ti ameliorate induced implant bone destruction in DM rats

**Fig. 7:**
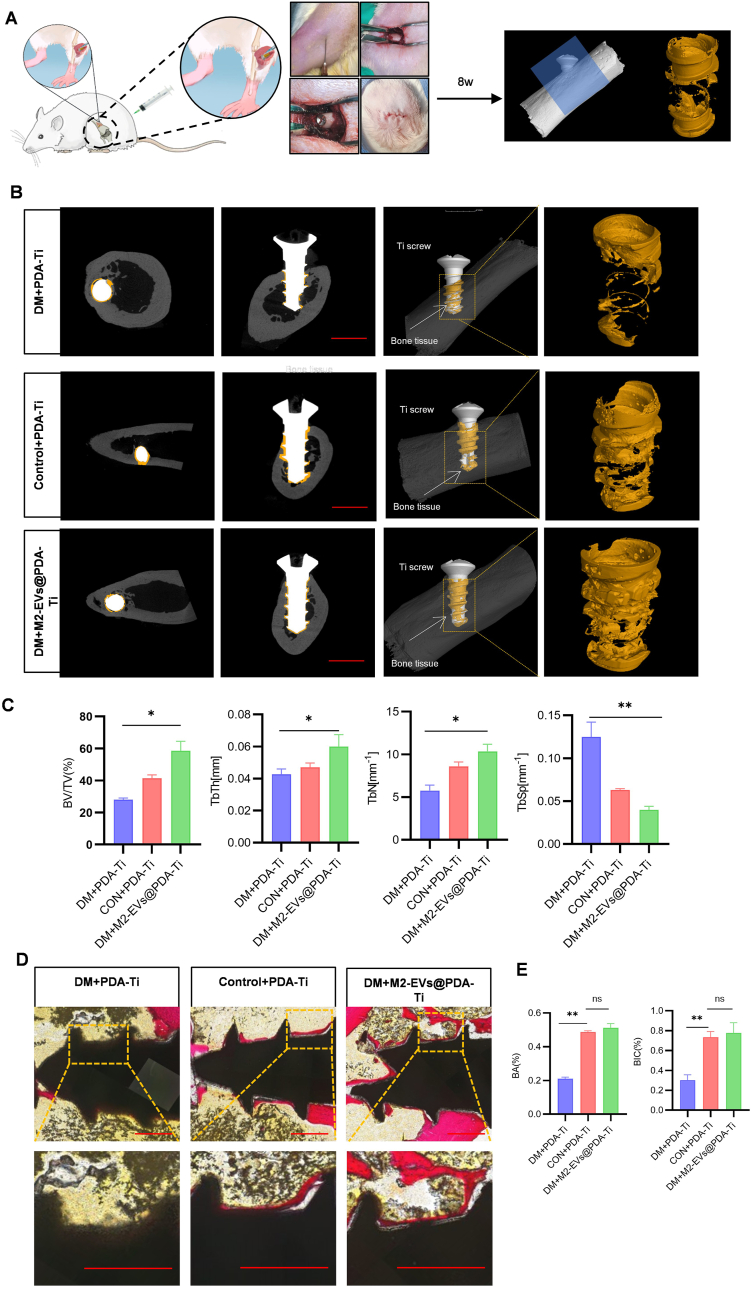
M2-EVs@PDA-Ti ameliorates induced bone destruction in DM rats. (A) Schematic of in vivo M2-EVs@PDA-Ti implantation process. (B) Micro-CT images of rat femurs, and three-dimensional reconstructed structures of the ROI, with BIC areas highlighted in yellow. (C) Analysis of histomorphometric parameters including BV/TV, Tb.Th, Tb.N, and Tb.Sp. (D) Van Gieson staining for histological evaluation of osseointegration and new bone formation around implants using (scale bar: 500 μm). (E) Quantification of BA and BIC ratio (n = 3, ∗*P* < 0.05, ∗∗*P* < 0.01). (For interpretation of the references to color in this figure legend, the reader is referred to the Web version of this article.)

Next, we performed in vivo experiments in DM rats to assess whether M2-EVs@PDA-Ti improved bone growth around the implants (Fig. 7A). We implanted Ti implants into rat femurs, and after 8 weeks, used micro-CT to observe whether the new bone that formed. We rebuilt three-dimensional images of fresh bone surrounding the implant (i.e., yellow zone; Fig. 7B). Bone parameters such as BV/TV, Tb.Th and Tb.N were measured, and they revealed changes in bone amounts and structures in the ROI (Fig. 7C). We noted an increase in new bone growth on the implant surface in both the control and M2-EVs@PDA-Ti groups. BV/TV, Tb.Th, and Tb.N also exhibited significant increases, whereas Tb.Sp decreased to almost half its baseline value. Histological assessment through Van Gieson staining was used to evaluate tissue healing around the bone–implant interface. In all groups, we noted bone formation in the implant regions (depicted in red) (Fig. 7D). In contrast, the DM group exhibited sparse and interrupted new bone growth, whereas the DM M2-EVs@PDA-Ti group demonstrated enhanced new bone growth with improved bone volume and consistency on the surface. Moreover, the BIC ratio and BA further supported these observations, demonstrating favorable trends in the DM M2-EVs@PDA-Ti group (Fig. 7E). Therefore, these results confirmed that M2-EVs@PDA-Ti effectively modulated bone immunity and increased bone integrity, effectively improving implant osseointegration.

The present study, despite providing valuable insights, still possessed certain limitations and deficiencies in terms of clinical translation [42,43]. As torque could influence the retention rate of M2-EVs on the surface of titanium during implantation, further investigation into the impact of torque on M2-EVs is warranted. To address this, M2-EVs@PDA-Ti implants were implanted into rats and subsequently reversely removed. Scanning electron microscopy (SEM) was utilized to examine the residual M2-EVs on the surface of the Ti implants. The results revealed that following implantation, the Ti surface retained a ratio of 51.9 % of M2-EVs (Fig. S9A). However, it was crucial to acknowledge that the specific enumeration of M2-EVs could only be conducted through statistical analysis of EVs in local SEM images, with a cluster of M2-EVs counted as a single unit. This limitation may affect the accuracy and reliability of the data. Considering this constraint, cel-mir-54 had been successfully loaded into the purified exosomes via an electroporation-based method, as evidenced by fluorescence microscopy images (Fig. S9B). Comparing the levels of cel-miR-54 in M2-EVs@PDA-Ti with and without implantation, the PCR results demonstrated that the content of cel-miR-54 on the implanted Ti surface still remains at 57.3 % compared to the non-implanted Ti surface (Fig. S9C). This can be attributed to the nanoporous structure and PDA on the titanium surface, which provides a favorable environment for the adsorption and binding of M2-EVs.Although some M2-EVs on the surface may indeed be lost due to the initial torque, M2-EVs are nanoscale vesicles, and those situated within deep nanopores can still remain on the titanium surface [44].

## 4 Conclusions

Here, we explored the relationship between bone repair ability and macrophage polarization during bone growth surrounding implants under diabetic conditions. The experimental findings revealed that in a DM femoral implant rat model, an imbalance in the bone immune microenvironment significantly delayed bone regeneration, as indicated by aberrant increases in the M1 macrophages number and reductions in the M2 macrophages number. Notably, M2-EVs could influence the immune response by reshaping M1 macrophages, reinstating equilibrium in the M1–M2 macrophage balance and thus fostering bone formation. Moreover, miR-23a-3p, targeting NEK7, represents a promising modality for partially reprogramming M1 macrophages through inhibition of NLRP3 inflammasome activation under diabetic conditions. These results provide a fresh perspective and potential therapeutic strategy for leveraging M2-EVs to enhance the Ti implant–bone immune microenvironment under hyperglycemic conditions.

## CRediT authorship contribution statement

**Yuzhao Cheng:** Writing – review & editing, Writing – original draft, Methodology, Investigation, Formal analysis, Data curation, Conceptualization. **Xin Dong:** Resources, Funding acquisition. **Jing Shi:** Formal analysis, Data curation. **Guangsheng Wu:** Visualization, Validation, Funding acquisition. **Pei Tao:** Formal analysis, Data curation. **Nan Ren:** Funding acquisition. **Yimin Zhao:** Formal analysis, Data curation, Conceptualization. **Fenglan Li:** Funding acquisition. **Zhongshan Wang:** Writing – review & editing, Writing – original draft, Project administration, Funding acquisition, Conceptualization.

## Availability of data and materials

The authors declare that the main data supporting the finding of this study are included in this published article and its additional elf information.

## Ethics approval and consent to participate

The experimental animal procedure was conducted under strict supervision and approved by the Fourth Military Medical University's Ethics Committee for the School of Stomatology. All experimental protocols followed ARRIVE guidelines. All participants consented to publish the paper.

## Consent for publication

All participants consented to publish the paper.

## Funding

This study was financially supported by the 10.13039/100014717National Natural Science Foundation of China grant (No.81700930),and the 10.13039/100017445Natural Science Foundation of Shandong Province(No.ZR2022MH062),the 10.13039/100012551Applied Basic Research Program of Shanxi Province(No.201901D111442),Shaanxi Provincial Key Research and Development Program(No.2022SF-521),the International Scientific and Technological Cooperation and Exchange Program in Shaanxi Province of China (NO. 2022 KW-17),the State Key Laboratory of Oral & Maxillofacial Reconstruction and Regeneration(No. 2020ZB01)

## Declaration of competing interest

The authors declare that they have no known competing financial interests or personal relationships that could have appeared to influence the work reported in this paper.

## Data Availability

No data was used for the research described in the article.
