# Supplementary material for: Immunomodulation with M2 macrophage–derived extracellular vesicles for enhanced titanium implant osseointegration under diabetic conditions
Source: Mater Today Bio. 2024 Dec 5;30:101385. doi: 10.1016/j.mtbio.2024.101385 (PMC11683253; doi:10.1016/j.mtbio.2024.101385)
Supplement: Multimedia component 1 [file mmc1.docx]

**Supplementary information**

**Immunomodulation with M2 macrophage–derived extracellular vesicles for enhanced titanium implant osseointegration under diabetic conditions**

Yuzhao Cheng^a,b,1^, Xin Dong^c,1^,Jing Shi^a,1^, Guangsheng Wu^b,d^, Pei Tao^b,e^,Nan Ren^b^, Yimin Zhao^b,*^, Fenglan Li^a,**^, Zhongshan Wang^b,***^

^a^The Stomatology Department of Shanxi Provincial People Hospital,Shanxi Medical University, Taiyuan, Shanxi 030001, China;

^b^State Key Laboratory of Oral & Maxillofacial Reconstruction and Regeneration,National Clinical Research Center for Oral Diseases,Shaanxi Key Laboratory of Stomatology, Department of Prosthodontics,School of Stomatology,The Fourth Military Medical University.

^c^Department of Orthopedic Surgery, Tangdu Hospital, Air Force Military Medical University, Xi'an, Shaanxi Province, China.

^d^Department of Stomatology, Qingdao Special Servicemen Recuperation Center of PLA Navy, No.18 Yueyang Road,Qingdao 266071, China.

^e^College of Chemistry and Bio-engineering, Yichun University,Yichun,Jiangxi 336000, China.

Correspondence should be addressed to:

* Corresponding author. State Key Laboratory of Oral & Maxillofacial Reconstruction and Regeneration,National Clinical Research Center for Oral Diseases,Shaanxi Key Laboratory of Stomatology,Department of Prosthodontics,School of Stomatology,The Fourth Military Medical University;

** Corresponding author. The Stomatology Department of Shanxi Provincial People Hospital,Shanxi Medical University, Taiyuan, Shanxi 030001, China;

*** Corresponding author.State Key Laboratory of Oral & Maxillofacial Reconstruction and Regeneration,National Clinical Research Center for Oral Diseases,Shaanxi Key Laboratory of Stomatology,Department of Prosthodontics,School of Stomatology,The Fourth Military Medical University;

**Supplementary Tables**

**TableS1.** The primers of genes used in the article.

| INOS-F | CAAGCACATTTGGGAATGGAGA |
| --- | --- |
| INOS-R | CAGAACTGAGGGTACATGCTGGAG |
| CD206-F | AGAGCTGGCGAGCATCAAGAG |
| CD206-R | TTCCATAGGTCAGTCCCAACCAA |
| ARG1-F | AGCTCTGGGAATCTGCATGG |
| ARG1-R | ATGTACACGATGTCTTTGGCAGATA |
| GAPDH-F | TGTGTCCGTCGTGGATCTGA |
| GAPDH-R | TTGCTGTTGAAGTCGCAGGAG |
| NLRP3-F | CTGAAGCATCTGCTCTGCAACC |
| NLRP3-R | AACCAATGCGAGATCCTGACAAC |
| IL-1β-F | CCCTGAACTCAACTGTGAAATAGCA |
| IL-1β-R | CCCAAGTCAAGGGCTTGGAA |
| Caspase-1-F | ACTCGTACACGTCTTGCCCTCA |
| Caspase-1-R | CTGGGCAGGCAGCAAATTC |
| TGF-B1-F | CATTGCTGTCCCGTGCAGA |
| TGF-B1-R | AGGTAACGCCAGGAATTGTTGCTA |
| NEK7-F | TCTTGGACTTGGTCGGTTCTT |
| NEK7-R | ACGCCGCCATCTCATACAG |
| miR-23a-3p | ATCACATTGCCAGGGATTTCC |
| OCN-F | CCCTCTCTCTGCTCACTCTGCT |
| OCN-R | CTTACTGCCCTCCTGCTTGG |
| OSX-F | ACCCCACCTCTTGCAACCAG |
| OSX-R | GTTTGCCTGCACCACTCCC |
| OPN-F | CCAGCCAAGGACCAACTACA |
| OPN-R | CTGCCAAACTCAGCCACTTTC |

**TableS2.** The antibodies used and the corresponding dilutions

| iNOS | 1:1000 | ab178945; Abcam |
| --- | --- | --- |
| CD206 | 1:1000 | #24595; Cell Signaling Technology |
| CD63 | 1:1000 | ab217345; Abcam |
| TSG101 | 1:1000 | ab125011; Abcam |
| Calnexin | 1:250 | ab133615; Abcam |
| GAPDH | 1:10000 | ab181602; Abcam |
| IL-1β | 0.2ug/ml | ab9722; Abcam |
| NEK7 | 1:10000 | ab133514; Abcam |
| caspase-1 | 1:1000 | #4199; Cell Signaling Technology |
| NLRP3 | 1:1000 | ab270449; Abcam |

**Supplementary Figures**

**Fig.S1Bone Marrow Mesenchymal Stem Cells (BMSCs) Identification by FCM.**


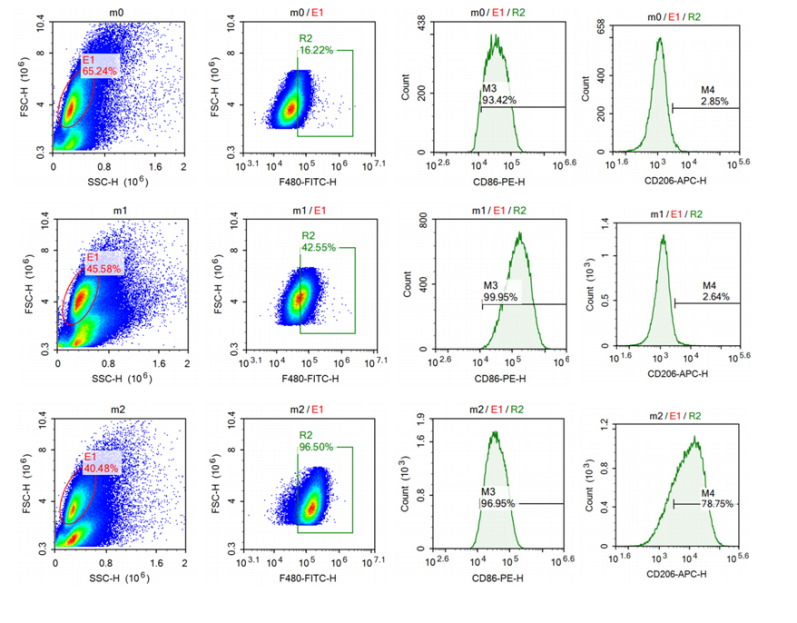


**Fig.S2 Percentage of CD86、CD206 positive M0、M1、M2 macrophages detected by FCM.**

**Fig.S3 Diabetes is detrimental to peri-implant osteogenesis**


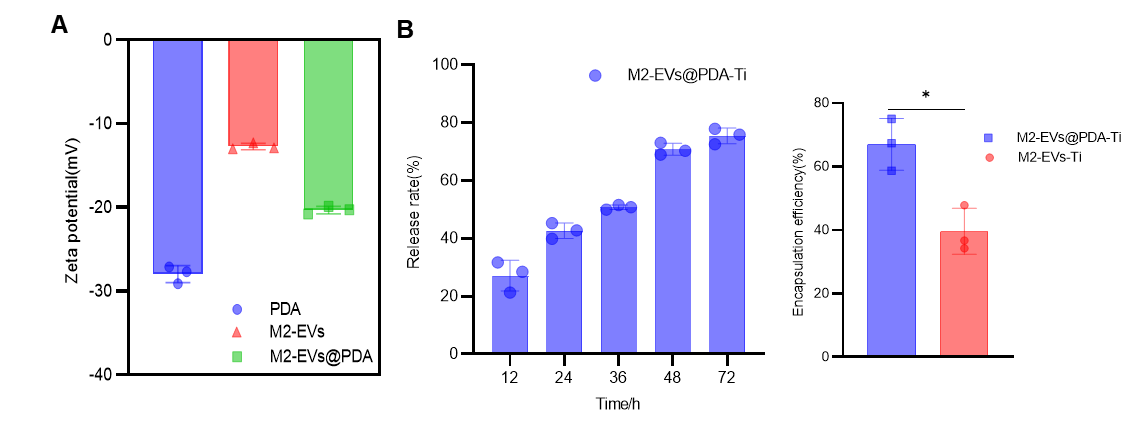


**Fig.S4 Estimation of M2-EVs coating efficiency PDA@Ti.**(A)Zeta potential measurements of PDA, M2-EVs, and [M2-EVs@PDA. (B)M2-EVs](mailto:M2-EVs@PDA.(B)M2-EVs) cumulative release efficiency of M2-EVs@PDA-Ti(n = 3; *P < 0.05)


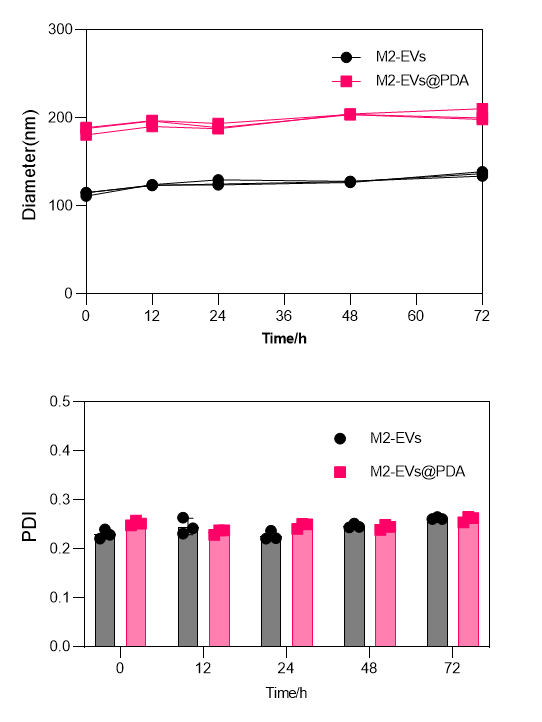


**Fig.S5 Detection of the stability of M2-EVs，M2-EVs@PDA.**


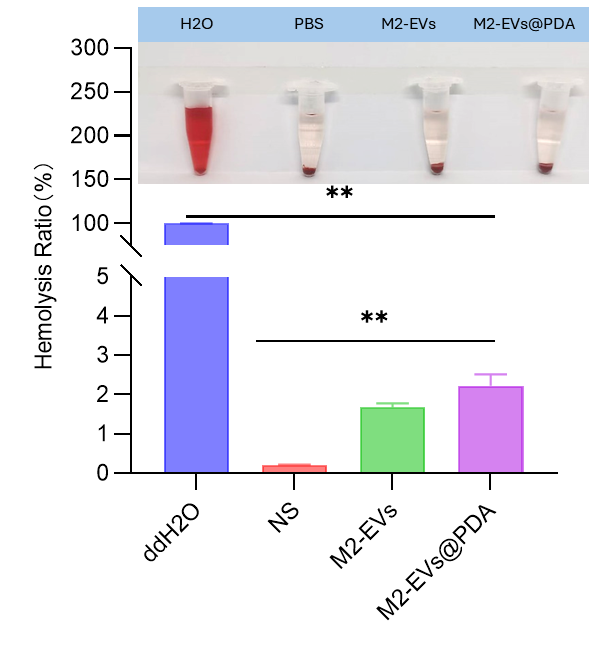


**Fig.S6 Schematic diagram of hemolysis assay results.**


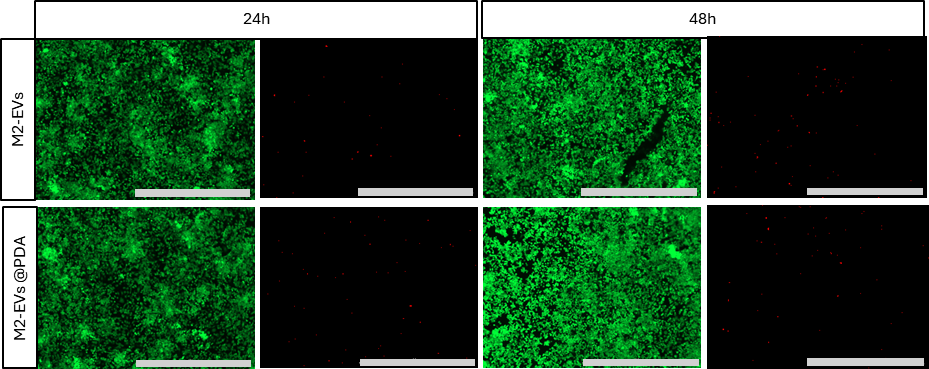


**Fig.S7 Live−dead of Macrophages treated with different times(24h\48h) of M2-EVs\M2-EVs @ PDA**


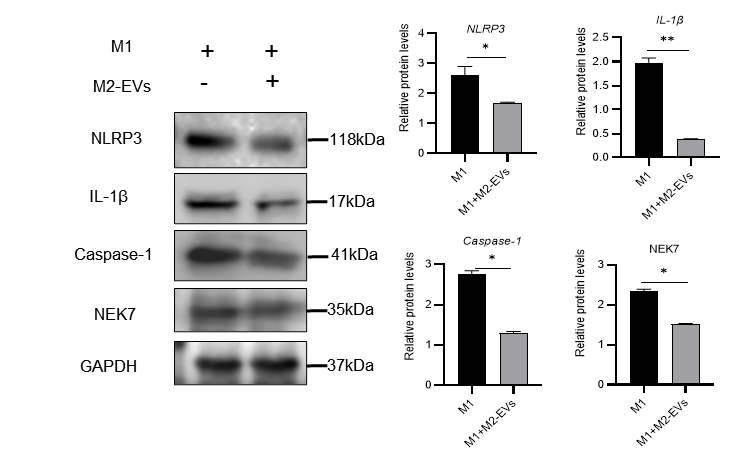


**Fig.S8** **Western blotting revealing that M2-EVs suppressed NLRP3, NEK7, caspase-1, and IL-1β production in M1 macrophages (n = 3; *P < 0.05, **P < 0.01).**


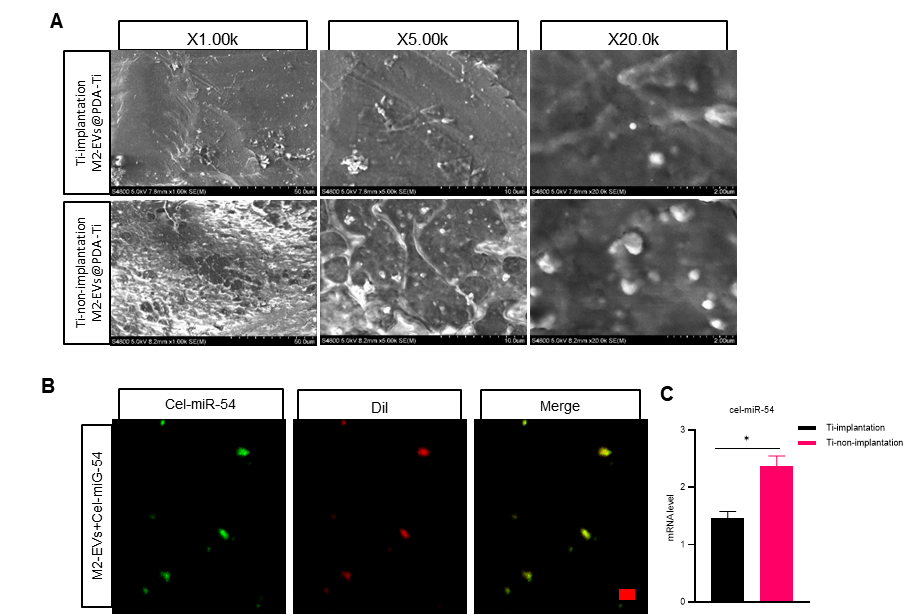


**Fig.S9 Retention of M2-EVs on the coating surface after implantation**(A)Surface topography images of M2-EVs@PDA-Ti after implantation (scale bar: 50, 10, or 2 µm). (B)cel-mir-54 had been successfully loaded into M2-EVs under a confocal microscope (scale bar: 10 μm).(C)qRT-PCR analysis Cel-miR-54 mRNA levels in M2-EVs@PDA-Ti with implantation and without implantation(n = 3; *P < 0.05).
